# Online adaptive E-tutorials for development of critical appraisal skills

**DOI:** 10.3389/fphar.2026.1940433

**Published:** 2026-10-01

**Authors:** Loyola McLean, Samuel Lane, Erick C. N. Wong, Louise Nash, Eryn L. Werry

**Affiliations:** 1 The Brain and Mind Centre, The University of Sydney, Sydney, NSW, Australia; 2 Specialty of Psychiatry, Faculty of Medicine and Health, The University of Sydney, Sydney, NSW, Australia; 3 School of Chemistry, Faculty of Science, The University of Sydney, Sydney, NSW, Australia; 4 School of Medical Sciences, Faculty of Medicine and Health, The University of Sydney, Sydney, NSW, Australia; 5 Sydney Medical School, Faculty of Medicine and Health, The University of Sydney, Sydney, NSW, Australia

**Keywords:** adaptive e-tutorials, animations, critical appraisal, graduate skill development, pharmacology education

## Abstract

Critical appraisal of research literature is an important skill for graduates yet science graduate employers report dissatisfaction with skill levels. As scaffolding can assist in critical appraisal development, we aimed to develop and evaluate 2 adaptive e-tutorials for critical appraisal development of neuropharmacology research articles as part of the scaffolding process. We developed adaptive e-tutorials allowing personalised feedback and remediation. Results of an end-of-tutorial survey, and comparisons of institutional Unit of Study Survey ratings and exam results in the 2 years before, and during, the intervention were analysed. Over 4 years, 150 students participated. In the cohorts that did not complete the e-tutorial, there were significant differences in exam marks between students of different academic backgrounds (non-science < science < medicine backgrounds; p < 0.05), while no significant differences between groups were seen in students that completed the e-tutorial, and these marks were statistically equivalent. Students ranked their perceived benefit from the videos, increase in critical appraisal ability, and value of the e-tutorial with average rankings between 4.4–4.6 on a Likert scale (5 = strongly agree). Similarly, students who completed the e-tutorial showed a significant increase in rating of their critical thinking skills in the Unit of Study Survey (p < 0.05). Completion of the e-tutorial was associated with increased equitable development of critical appraisal. Students from non-science backgrounds showed the biggest increase in actual appraisal performance, suggesting that while the e-tutorial was associated with increased confidence for all students, the other features of the course may have developed appraisal levels to a stage sufficient for successful exam completion.

## 1 Introduction

Critical thinking, including critical appraisal of research literature, is an essential competency for pharmacology graduates across clinical, research, and industry domains. Critical thinking has been identified as a ‘future-proof’ graduate skill in the face of transformational pressures from growing artificial intelligence use in the workforce, with the ability to critically appraise output of large language models integral to its productive use (Du et al., 2023). Modelling based on 20 billion hours of work data by the Foundation for Young Australians (2017) predicted that workers will spend 41% more time in critical thinking by 2030 (Foundation for Young Australians, 2017). A 2026 survey by the National Association of Colleges and Employers in the USA found 93.5% of employers rated critical thinking as an essential competency they sought for graduate positions in their company (National Association of Colleges and Employers, 2026). This trend is also evident across the sciences, with 128 Australian science, technology, engineering and mathematics (STEM) employers reporting critical thinking was one of their top-valued graduate employee attributes, with 95% agreeing or strongly agreeing it was highly valued (Rayner and Papakonstantinou, 2015). It is, therefore, no surprise that the Education Working Group of the European Association for Clinical Pharmacology and Therapeutics included critical appraisal as one of their core learning outcomes (Brinkman et al., 2018).

Despite this, Arum and Roska have shown there were no significant gains in critical thinking ability across the first 2 years of university in the 2,300 U.S. students assessed using a Collegiate Learning Assessment (Arum and Roksa, 2011). This is reflected in a survey of 185 US employers, which revealed only 50% of graduate employees were rated as competent in critical thinking (National Association of Colleges and Employers, 2026). This trend is upheld with science graduates, where a survey in Australia found 23% of science graduate employers were dissatisfied with the critical thinking abilities of new science graduates (Evolve Scientific Recruitment, 2016).

One process that can facilitate critical thinking development is scaffolding, where structured support is initially provided for students, for example, in the form of modelling critical appraisal skills. Students are then provided opportunities to practice appraisal with support mechanisms that are gradually removed when proficiency increases and students develop greater independence (Persky et al., 2019; Holmes et al., 2015). Journal clubs, where peers discuss and evaluate a journal article with an instructor, is one form of scaffolding that has shown utility in developing critical appraisal in pharmacology postgraduate students (Gajbhiye et al., 2021). However, journal clubs have been a feature of pedagogy for decades and the aforementioned graduate employer dissatisfaction of critical thinking has persisted, suggesting further scaffolding activities may have added value.

Interactive e-learning tools have shown efficacy in improving learning across a range of disciplines, including for health professional and medical science cohorts (Wimalarathna et al., 2021; Jayawardena et al., 2024; Kulik and Fletcher, 2016). Adaptive e-tutorials are a subtype of tool that utilise software or applications to deliver learning activities in a manner that allows provision of feedback, and supply of remediation or extension, tailored to the needs of individual students. They offer advantages to in-class activities such as journal clubs, by allowing students to practice critical appraisal in their own time, in a self-guided manner, with feedback targeted to each of their critical appraisal decisions. It provides an extra layer of scaffolding, and has shown utility in developing critical thinking in other disciplines, including English literacy (Yang et al., 2013). The individualisation of this approach has also helped with equitable outcome achievement across learners with differences in initial starting knowledge in a cytopathology postgraduate degree unit (Van Es et al., 2015). The utility of adaptive e-tutorials for the development of critical thinking in pharmacology research appraisal skills amongst university students has not yet been assessed, nor has their ability to facilitate equitable outcomes in the achievement of these skills. Accordingly, our study aimed to design an adaptive e-tutorial for the individualised practice of pharmacology research critical appraisal and evaluate its impact on acquisition of this skill amongst a cohort with diverse academic backgrounds.

## 2 Methods

### 2.1 Study design

This study aligns with a quasi-experimental design, where true randomisation to intervention and control groups was not possible, providing a middle ground between observational studies and experimental clinical trials (Capili and Anastasi, 2024). It applies a ‘post-test only with a control group’ subtype of quasi-experimental design. The design follows the Transparent Reporting of Evaluations with Non-Randomised Designs (TREND) guidelines (Des Jarlais et al., 2004). It also follows a mixed-methods design, using both quantitative exam results, end-of-semester institutional unit of study survey results and qualitative content analysis of a survey administered at the end of the second e-tutorial.

### 2.2 Study population and setting

Participants consisted of students in the ‘Research Inquiry (BMRI5020)’ unit at the University of Sydney across 4 cohorts (2017–2020). This unit is a 6-credit point core unit in both the 1-year postgraduate coursework Master of Brain and Mind Sciences and the 3-year part-time Master of Medicine (Psychiatry) at the University of Sydney, with second year students in this degree taking the subject. Research Inquiry is designed to equip students with the critical appraisal skills to read widely across health and medical science literature and judge which findings are reliable for informing future research and clinical practice.

In its original form (2017–2018), Research Inquiry featured 2 h weekly lectures every week of a 13-week semester. Lectures were on aspects of good research design, focussing on a different study type pertinent to clinical and basic pharmacology each week, especially neuropharmacology (e.g., case-control studies, clinical trials, qualitative methods, animal studies, electrophysiology and *in vivo* pharmacology, and methods relating to proteins and genes). In 5 of these lectures, around 30 min were dedicated to a journal club. Students had to submit answers to critical appraisal questions on the journal club article before the lecture and these were discussed within the lecture, facilitated by the lecturer. The submitted answers were worth 2% of the total mark per journal club and were marked on submission rather than accuracy. At the end of semester, students completed a final open book 120 min exam worth 45% of their overall mark. It was designed to assess student’s ability to critically appraise a neuropharmacology paper (e.g., probing their ability to identify risk for different biases and confounds and whether they were adequately controlled for, whether correct statistics and research design were chosen and why, whether conclusions were sound, and whether there were conflicts with existing literature). This was how the subject was engaged in by the initial 2 cohorts.

The 2 cohorts that completed the e-tutorials (2019–2020) engaged in the same lectures and journal clubs as the 2 earlier cohorts which were not offered the e-tutorials. They also completed the same end-of-semester exam with the same questions on the same journal article. In addition, in week 7 and week 13 (3 weeks before the end-of-semester exam), they completed the adaptive e-tutorials described below.

Teaching staff stayed largely the same between cohorts. Out of the 16 staff members involved each semester, only 4 changes occurred between 2017 and 2018, no changes occurred between 2018 and 2019 and 1 change occurred between 2019 and 2020. Lecture material was reviewed for sessions with staff changes and no systemic differences were identified.

### 2.3 Educational intervention–adaptive E-tutorials

The 2 adaptive e-tutorials were developed using Smart Sparrow’s Adaptive eLearning Platform. This is a platform that hosts content and the ability of someone to navigate through that material can be directed using a rule-based platform (e.g., if an initial question is answered correctly, the person is directed to second screen that has a follow up question to check the correct reasoning was used to get the answer, whereas if the initial question is answered incorrectly, the person is directed to a screen showing remediation material). This platform was chosen as other Smart Sparrow adaptive e-tutorials have shown efficacy in directing practice to where it is needed, providing more personalized and equitable learning opportunities, and improving performance, confidence and engagement (Van Es et al., 2015; Samulski et al., 2016).

Each adaptive e-tutorial had three stages (Supplementary Figure S1).

1. Introduction. Both e-tutorials begin by outlining the importance of critical appraisal (e.g., where they may use it in a range of potential future jobs and a real-life example about what can go wrong if it is not done well) and the learning objectives.
2. Key concept familiarisation: The second stage featured multiple choice questions, which allowed students to assess their understanding of the main concepts (e.g., internal validity, external validity, reliability, face validity, predictive validity, construct validity). Using Smart Sparrow’s question trap state feature which allows programming of progression direction dependent on answers to a question, if they got a question correct then they moved to the next concept. If they did not get a question correct, they were directed to personalised feedback and remediation materials, including a suite of 8 animated videos we developed. Animated videos were chosen for concept remediation as studies from several disciplines found they can be effective at helping students master concepts (Barak et al., 2010; Hwang et al., 2012; Taylor et al., 2007). Informal conversations with students also suggested that they regularly engaged with animated content for recreation and found critical appraisal concepts challenging, indicating animated videos may help make them concepts more approachable. These videos were developed using the software Vyond and are housed on Youtube at: https://www.youtube.com/@DrEW-oc3ou
3. Application and practice: In each e-tutorial, students were instructed to read the methods, results and conclusions of a study we wrote. For the first e-tutorial, this was a clinical trial for a new drug. For the second e-tutorial, this was a preclinical drug data package for a new biotech startup that was requesting funding. Each study contained examples of both good and flawed design and interpretation. Students had to click on each sentence they thought was problematic to highlight it. If they correctly highlighted a flawed sentence, they received a follow up question to check that they had used the correct reasoning, and that they understood the underlying problem. If they did not highlight a flawed sentence, they were taken to remedial information and a follow up question to ensure they understood, before returning them to the initial study where they were asked to identify that particular flaw again.

The first e-tutorial examined good clinical trial study design and the second examined good design of common techniques used in preclinical pharmacology lab publications (e.g., animal studies, immunofluorescence, genetic manipulation). Completion of each e-tutorial (regardless of accuracy) was worth 5% of the overall mark for the unit.

### 2.4 Data collection and statistical analyses

The collected outcome measures were.

- An end-of-tutorial survey (8 Likert scale questions, listed in Figure 1, 2 open-ended questions (‘What can we do to improve the e-tutorial?’ and ‘The aspect of the e-tutorial I found most beneficial was….‘?).
- Comparison of average ratings on the end-of-semester Institutional Unit of Study Survey from the 2 years before the intervention and the 2 years during the intervention. We were supplied with average, variance and sample size from each year which was used to compare pooled averages and variances from 2017–2018 with 2019–2020 using a Student’s t-test (p < 0.05 was considered as significantly different).
- A comparison of average marks in the end of semester exam between students of different academic backgrounds in the 2 years before the intervention compared to the 2 years during the intervention, using a one-way ANOVA with Sidak’s *post hoc* analysis and Welch’s equivalence tests (p < 0.05 was considered as significant). This exam asks students to critically appraise a neuropharmacology-based article. It was a 120 min partial open-book exam, where students could bring in the journal article with notes on front and back. Exams were marked in a blinded fashion by 3 markers, with inter-rater reliability of 0.79. Equivalence bounds of 20 were used in the Welch’s equivalence tests. 20% in the exam represents ∼4 questions out of the total examination of 20 questions. Each of the 20 questions probes a different aspect of critical appraisal, so a difference in 20% represents a difference in mastery or application of ∼4 critical appraisal concepts. Differences smaller than this margin were judged unlikely to represent a practically meaningful difference in the overall critical appraisal skills of learners and overall judgement of the paper’s validity and were, therefore, considered equivalent.

**FIGURE 1:**
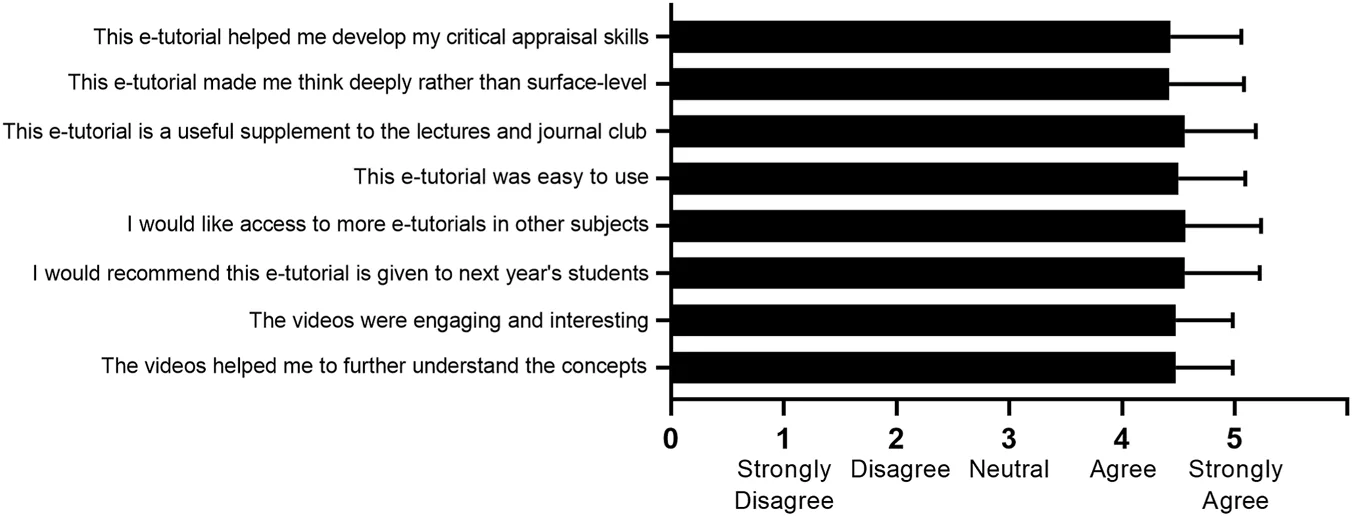
Perceived benefit of the critical appraisal e-tutorial using a Likert scale. Values are mean ± standard deviation (n = 32).

### 2.5 Content analysis

De-identified survey responses to the first 2 open answer questions of the post-e-tutorial survey were analysed. They asked about suggestions for improvement and the most beneficial aspect of the e-tutorial. There were 32 text responses to each of these questions. The background theory utilized was that adult learners are a good judge of their needs for teaching and learning and their reception and review of the resource is valuable feedback (Knowles, 1984a; Knowles, 1984b).

The content analysis proceeded in a step-wise fashion (Hsieh and Shannon, 2005). After immersion in the data with reading and rereading, beginning at the level of the paragraph and moving down to the word level, considering semantic meaning, trial codes were assigned and then checked for inclusion of all the data set, with each question analysed separately. An experienced qualitative coder (LM) undertook the first pass analysis with results then shared for consideration and review with the resource designer (EW).

### 2.6 Ethics approval and recruitment

This study was approved by the University of Sydney Human Research Ethics Committee (2019/103). Exam and Unit of Study Survey data from the 2 cohorts before inclusion of the adaptive e-tutorials were obtained for all students under an approved waiver of consent. For the 2 cohorts that completed the e-tutorials, students read a participation information sheet before completion and could opt-in to having their de-identified survey responses and exam marks included in analysis *via* a participant consent form. Exclusion criteria for participants who opted in during the 2 e-tutorial cohort years was non-completion of one or both e-tutorials. No students met this exclusion criteria.

## 3 Results

### 3.1 Participants

Over 4 years, 150 students participated, with demographics summarised in Table 1. As is common to many postgraduate degrees, students had a wide variety of undergraduate or previous postgraduate academic backgrounds, including students who had completed medical degrees, biological science-related degrees (e.g., Bachelor of Science, Psychology, Medical Science, Biotechnology, Psychological Science or Health Science) and students from a non-biological science background (e.g., Bachelor of Communication, Arts, Special Education, Commerce, Education, Engineering, International and Global Studies).

**TABLE 1:** Background of participants.

Academic background	Without e-tutorial n (% of group that participated)	With e-tutorial n (% of group that participated)
Non-biological science	26 (100%)	15 (62.5%)
Biological science	27 (100%)	20 (91%)
Medicine	44 (100%)	18 (55%)
Total	97	53

### 3.2 Survey after the second e-tutorial

#### 3.2.1 End of E-tutorial survey results–Likert scale questions

Questions on an end-of-tutorial survey probed student’s perceived increase in critical appraisal ability, useability and whether they thought the e-tutorial was a valuable addition to the course (Figure 1). 32 of 54 participants responded (59%). The average ranking on a Likert scale given in response to all questions was between 4.4–4.6 (1 = strongly disagree, 5 = strongly agree; Figure 1).

All students either agreed, or strongly agreed, that the animated videos were interesting and engaging, and helped them to further understand relevant concepts (Figure 1). Since their initial upload in December 2018, there have been over 220,000 total views on YouTube, with two of the videos viewed over 123,000 and 96,000 times. A YouTube traffic source analysis showed that over 50 universities have embedded, or provided a link to, some of these videos on their university e-learning platforms.

#### 3.2.2 End of E-tutorial survey results–Open answer questions

##### 3.2.2.1 Question 1. What can we do to improve this e-tutorial?

Valence of the evaluation of the e-tutorial as a whole

Results of the qualitative analysis are summarized in Supplementary Table S1. Of the 32 responses, 11 (34%) provided a positive evaluation of the e-tutorial as a whole, either through a clear positive statement or by saying there were no suggestions to make to improve it. At times both a positive evaluation and suggestion for improvement were offered, e.g.,: *“More videos, even if the question was right, they really helped with explaining, very succinct, clear and concise. Very 'to-the-point’ and a powerful learning tool”.* Two of the responses suggested expanding the e-tutorials, with one suggesting it be used as an opportunity to summarize the whole course for revision purposes: “*…given its the revision one before exams would use as opportunity to summarise the whole course*.”

Only one respondent (3%) gave a clear negative evaluation but moderated this with a suggestion to split the tutorial into 2 parts: *“I found the e-tutorial really difficult, it may have been better to break it into 2 parts”.*

Evaluation of aspects of the e-tutorial
  Videos

Four of the responses (13%) explicitly valued the teaching videos within the tutorial, suggesting more of them be used. One of these responses further suggested that there be more visual learning resources and less text as a whole: “*More videos/diagrams and less text.*”

Practice Questions

Four of the responses (13%) valued the question task explicitly, suggesting more examples and questions, e.g., *“Providing practice questions and more examples of different validities, reliabilities and research protocol problems in different scientific contexts.”*

Explanations

Three respondents (9%) suggested more explanations in the tutorial but varied as to where that should apply: one suggested more detailed explanation of principles; another suggested more explanation of the answers; the third suggested more explanation of types of statistical tests used. A fourth respondent found that answers offered in the appraisal task lacked clarity, finding some answers “vague”, implying that improved clarity would be beneficial.

Traumatic response to details of animal behavioural studies

One respondent (3%) considered the study examples included aspects of animal abuse and found them horrendous, noting that their inclusion was “unnecessary”: *“We could learn these concepts without such study examples.”*

Improving the learning pathway
  Technical capabilities and flow of the tutorial

Seven respondents (22%) suggested increasing the flexibility in the learning pathway by allowing a “back button” for review, or flexible movement back and forward between parts of the tutorial, without needing to restart it, with one further suggesting providing “*a link to the videos and text of tutorial for further review*.” Another suggested showing how many slides were left, *“to help track the progress through [the tutorial]”.*

Linking to other learning resources

Four respondents (13%) suggested greater linking to other learning resources. Three of these suggested that references be offered or hyperlinked to help learning and review. The fourth suggested *“more examples from the theory learned in lectures”.*

##### 3.2.2.2 Question 2: the aspect of the e-tutorial I found most beneficial was

Valence of the evaluation of the e-tutorial as a whole

Of the 32 responses to this question, 4 respondents (13%) gave a global positive evaluation of the e-tutorial, with these responses suggesting the helpfulness of the resource for revision and remediation: “*the whole thing was really useful*”; “*Love the e-tutorials overall!*”

Evaluation of aspects of the e-tutorial
  Videos

These were highly valued with 12 of the responses (38%) citing the videos as the most beneficial aspect or one of the beneficial aspects: *“And of course the videos. I strongly recommend that as much as possible videos be included.”*

The videos were valued for their explanations fostering understanding and their interest.

Appraisal Tasks

These were also highly valued with 14 of the responses (44%) citing this as the most beneficial aspect or one of the beneficial aspects. The idea of having to apply the knowledge, for example, in the section highlighting problematic sentences, was seen as useful, with corrective feedback following: “*Analysis of the sentences and highlighting the erroneous ones then going through them step by step the way the vocab was broken down*”; “*I like … getting to apply my knowledge (for example, highlighting the sentences that are problematic).*”

The Learning Pathway, Structure and Process

Eleven respondents (34%) cited the learning pathway, structure or process as the most beneficial aspect. This covered the flexible movement through sections; the way the learning journey started from basic concepts, and offered revision of definitions and concepts, and tested application of them, then using targeting of videos when concepts were not identified. The use of examples and clear explanations, with understandable notes was valued. One respondent valued the process of learning from their mistakes without stress:

“There is no stress about answering the questions. Mistakes are fine and allow you to have better understanding why you made them.”

One respondent expressed that: “It would have been nice if we could download the e-tutorial.”

Synthesis of the content from Questions 1 and 2

Collectively, the content of the responses suggest that this teaching resource, as a whole, was valued and felt useful to the students. The qualitative feedback supported the use of a careful learning pathway, building from simple to more complex, using videos and then active appraisal tasks, with opportunities for active application of knowledge and targeted correction of misunderstandings.

### 3.3 Institutional unit of study survey

Consistent with these results, there was a significant increase in student’s rating of their critical thinking skills in the University of Sydney’s end of semester Unit of Study Survey by those that completed the e-tutorial compared to those that did not (Figure 2A; *t*(54) = 2.64; Cohen’s *d* = 0.705 (95% Confidence Interval (CI) [0.17, 1.25]; p = 0.011). There was also a significant increase in the overall ratings for the subject (Figure 2B; *t*(54) = 2.39; Cohen’s *d* = 0.638 [95% CI: 0.09, 1.17]; p = 0.021).

**FIGURE 2:**
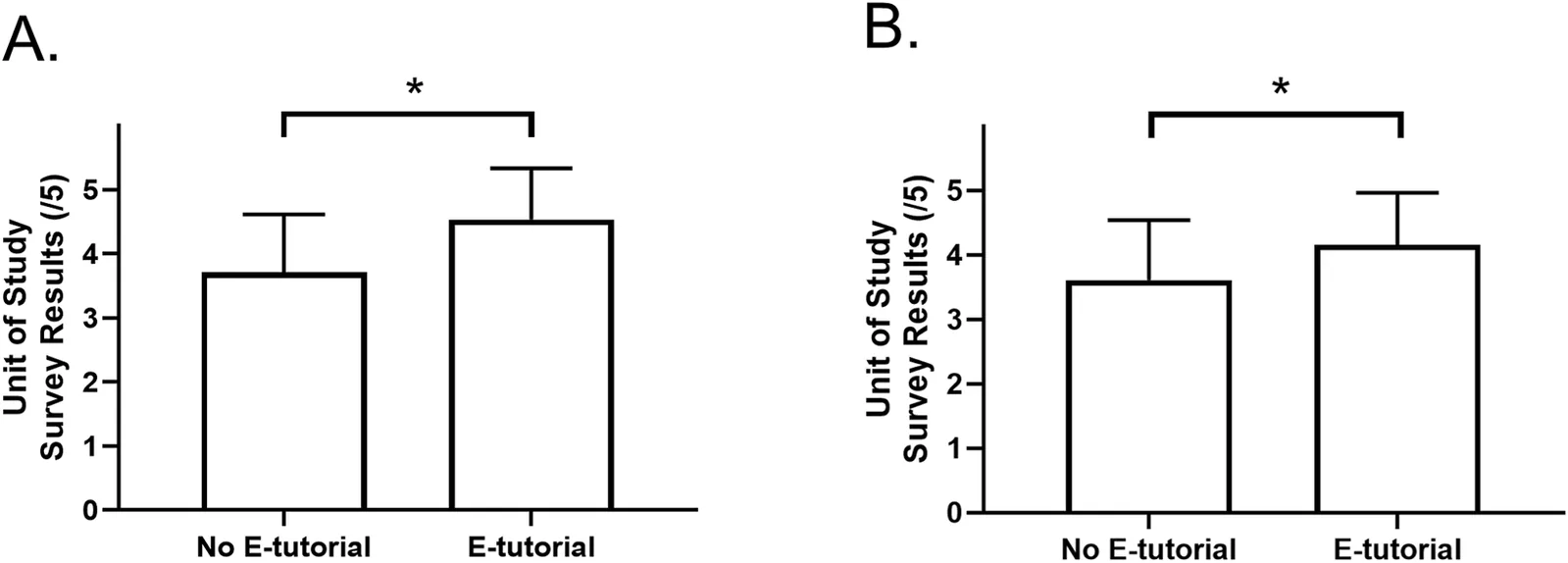
Average rating on the overall end-of-semester Unit of Study Survey for **(A)** the question ‘*I developed relevant critical and analytical thinking skills*’ and **(B)** the overall unit. *p < 0.05. Values are mean ± standard deviation (n = 28/group).

### 3.4 Exam marks

In the cohorts that did not complete the e-tutorial, there were significant differences in exam marks between all groups (Figure 3; non-science < science < medicine backgrounds; *F*(2, 95) = 19.86; p < 0.05). In the cohorts that completed the e-tutorial, there were no significant differences between groups (Figure 3; *F*(2,48) = 1.695; p > 0.05). In addition, in the cohorts that did not complete the e-tutorial, Welch’s equivalence tests found marks were not equivalent between students of non-science and medical backgrounds (Cohen’s *d* = 1.54, 90% CI [1.09, 2.10]), non-science and science backgrounds (Cohen’s *d* = −0.633, 90% CI [-1.13, −0.18]) and science and medical backgrounds (Cohen’s *d* = −0.90, 90% CI [-1.26, −0.40]). In contrast, a separate Welch’s equivalence test on the cohort who did complete the e-tutorial found exam marks were equivalent amongst students from non-science and medical backgrounds (Cohen’s *d* = −0.665, 90% CI [-1.34, −0.0831], non-science and science backgrounds (Cohen’s *d* = −0.31, 90% CI [-0.92, 0.26]) and science and medical backgrounds (Cohen’s *d* = −0.34, 90% CI [-0.93, −0.22]).

**FIGURE 3:**
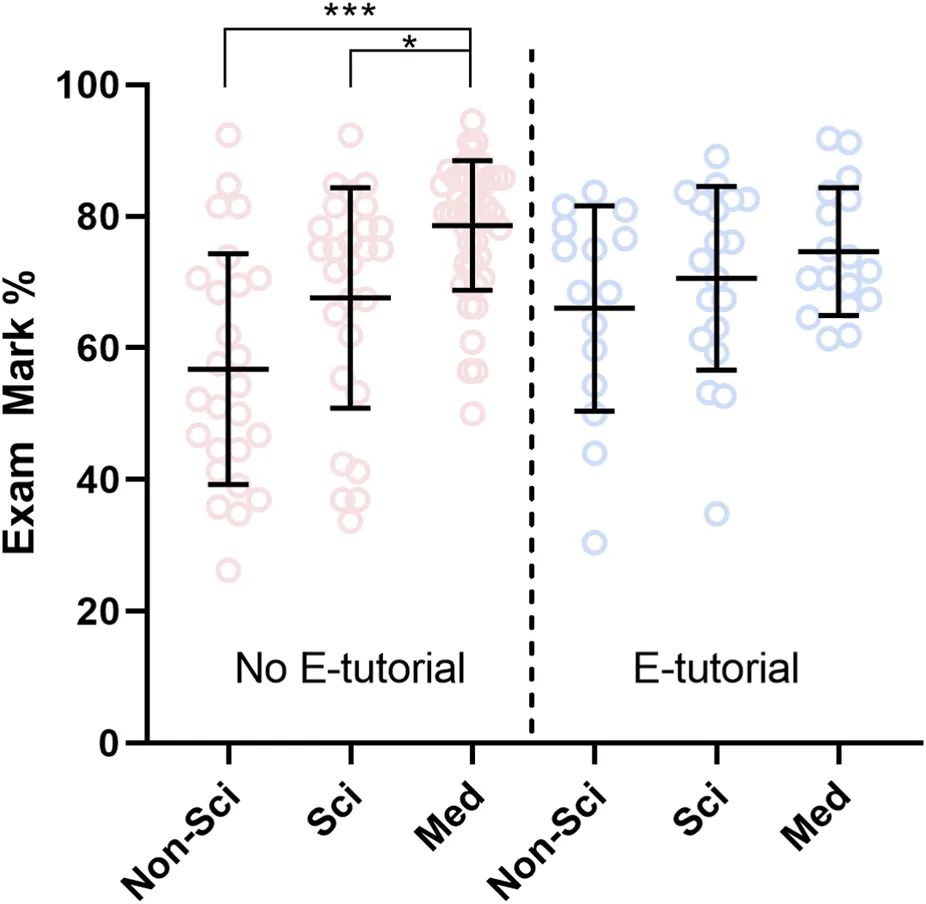
Average exam mark achieved by students from a non-science (Non-Sci), science (Sci) or medical (Med) undergraduate background, according to whether they completed the e-tutorial. The end-of-year exam probes student’s ability to critically appraise a neuropharmacology article. ***p < 0.001, *p < 0.05. Values are mean ± standard deviation, with n’s described in Table 1.

## 4 Discussion

Completion of the adaptive e-tutorials was associated with more similar outcomes across academic backgrounds in critical appraisal development. Although students from all backgrounds reported the e-tutorial was useful and indicated an increased confidence in their critical appraisal abilities, students from non-biological science undergraduate backgrounds demonstrated the biggest increase in exam marks. This suggests that while the e-tutorial may have increased confidence for all students, the other features of the course (e.g., lectures, journal clubs) may have already developed their critical appraisal levels to a stage where they were able to perform well in the exam. On the other hand, the tutorials were associated with increased support for learners less experienced in critical appraisal. These findings parallel other studies that report adaptive e-tutorials allow equitable acquisition of content (Van Es et al., 2015; Samulski et al., 2016), strengthening the utility of this approach for equitable education.

Key features of the adaptive e-tutorial valued by students include questions to check their understanding of basic concepts, an appraisal task allowing them to apply their knowledge to appraise research and offering tailored feedback and clear explanations in these activities. It was valued they could do this in a low-risk environment before attempting appraisal by themselves in the exam. In effect, this provides an individualised low-stakes method of progressive removal of scaffolding for critical appraisal development. This approach of allowing critical analysis practice with progressive removal of scaffolding is modelled on the method of Holmes *et al* (Holmes et al., 2015), which produced lasting improvements in student’s critical analysis of their own laboratory practical data. Our study has shown that removal of scaffolding also has utility for critical appraisal of research literature and can be done in a flexible and scalable manner.

This study also provided evidence that animated videos can be an approachable and engaging way of introducing core critical appraisal concepts and may increase students’ confidence in these concepts. This is supported by Mayer’s cognitive theory of multimedia learning, which posits that well-designed multimedia resources facilitate deeper understanding by integrating visual and verbal information (Mayer, 2012). Further to this, meta-analyses have also shown that animation can improve comprehension of complex scientific processes when aligned with instructional goals (Hoffler and Leutner, 2007). Importantly, in this study, videos were not used in isolation but embedded within an adaptive feedback loop.

Several considerations should be kept in mind when interpreting these findings. First, the quasi-experimental design without randomisation introduces the possibility of selection bias and cohort effects, meaning differences between groups cannot be definitively attributed to the intervention. For example, there were differences in participation rates across academic backgrounds, both within historical and intervention cohorts and between these cohorts. Without knowing if this represents a selection bias underpinned by differences in motivation or engagement, and without knowing the impact of the compulsory vs. self-selection recruitment between cohorts, we cannot rule out an influence of these on outcome comparisons. Similarly, we did not have access to data on demographic background (apart from academic background) or past academic performance so cannot rule out the influence of cohort differences between these factors on results. In addition to a potential influence of selection bias and cohort factors, the study relied on a single assessment modality (end-of-semester exam) to evaluate critical appraisal skills. While carefully designed, this may not fully capture broader or longer-term application of these skills. The use of self-reported survey data also introduces the potential for response bias, as students who engaged more positively with the tutorials may have been more likely to complete evaluations.

Future research should build on these findings through randomised controlled designs with adaptive *versus* non-adaptive formats to investigate causative links. Larger, multi-institutional studies would improve generalisability and allow exploration of how adaptive e-tutorials perform across diverse educational and cultural contexts (e.g., undergraduate students). Longitudinal studies are also needed to determine whether any associated changes in critical appraisal are retained over time and transferred to real-world contexts, such as research projects, clinical decision-making, or evaluation of artificial intelligence-generated outputs. Finally, future iterations of the e-tutorials could incorporate greater learner control, improved navigation, and additional linked resources, as suggested by student feedback, as well as expanded content to support revision and integration across the curriculum.

In summary, this study provides evidence that adaptive e-tutorials were associated with reduced differences in examination performance across academic backgrounds in postgraduate pharmacology education. By combining scaffolded learning, adaptive feedback, multimedia support and applied practice, and with further investigation of causal links, these tools may offer a promising, scalable approach to addressing persistent gaps in critical thinking development across diverse learner cohorts.

## Data Availability

The raw data supporting the conclusions of this article will be made available by the authors, without undue reservation.
