# Supplementary material for: Online adaptive E-tutorials for development of critical appraisal skills
Source: Front Pharmacol. 2026 Oct 1;17:1940433. doi: 10.3389/fphar.2026.1940433 (PMC13668495; doi:10.3389/fphar.2026.1940433)
Supplement: Supplementary file 1 [file Supplementaryfile1.docx]

**Online Adaptive E-tutorials for Development of Critical Appraisal Skills**

**Loyola McLean^1,2^, Samuel Lane**^3^**, Erick C.N. Wong^4^, Louise Nash**^1,2^**, Eryn L. Werry**^3,5 *^

1. The Brain and Mind Centre, The University of Sydney, Sydney, New South Wales, Australia

2. Specialty of Psychiatry, Faculty of Medicine and Health, The University of Sydney, Sydney, New South Wales, Australia

3. School of Chemistry, Faculty of Science, The University of Sydney, Sydney, New South Wales, Australia

4. School of Medical Sciences, Faculty of Medicine and Health, The University of Sydney, Sydney, New South Wales, Australia

5. Central Clinical School, Faculty of Medicine and Health, The University of Sydney, Sydney, New South Wales, Australia

*** Correspondence:**
Eryn Werry
eryn.werry@sydney.edu.au

Keywords: critical appraisal, adaptive e-tutorials, animations, pharmacology education, graduate skill development

**SUPPLEMENTARY FIGURE**

(A) Introduction about the importance of critical appraisal, and a story of what can happen when it goes wrong (Theranos) (4 screens – example below)

(B) Objectives (1 screen)

(C) Questions allowing students to check their understanding of the main concepts (eg internal validity, external validity, reliability, face validity, predictive validity, construct validity; 7 screens - example below). These were accompanied by personalised feedback and remediation materials, including animated videos, if their answers to questions indicated they were needed by the student (example below).

(D) Application and practice: students are asked to critically appraise a 3-part ‘Data Overview Package’ from a fictional neuropharmacology-based startup, with personalised remediation or positive feedback on their attempts. (19 screens; example of a task shown below)

(E) Summary screen, providing a checklist of questions students can use to critically appraise pre-clinical pharmacology articles, and further reading (1 screen)

Supplementary Figure 1. Overview of the neuropharmacology-based critical appraisal adaptive e-tutorial

**SUPPLEMENTARY TABLE**

Table S1. Summary of themes derived from end-of-etutorial open-ended survey responses.

| **Theme** | **Description** | **Illustrative Evidence** | **Prevalence** |
| --- | --- | --- | --- |
| Overall positive evaluation and perceived value | Students viewed the e-tutorial as useful, engaging, and valuable. | “The whole thing was really useful”; “Love the e-tutorials overall!” | Q1: 34%; Q2: 13% |
| Videos enhanced engagement and understanding | Videos helped explain difficult concepts and maintain interest. | “More videos...they really really helped with explaining” | Q1: 13%; Q2: 38% |
| Active learning through appraisal tasks | Application activities and feedback supported learning. | “I like… getting to apply my knowledge (for example highlighting the sentences that are problematic).” | Q1: 13%; Q2: 44% |
| Structured learning pathway supports perception of understanding | Scaffolded progression and low-stress learning were valued. | “Mistakes are fine and allow you to have better understanding...” | Q2: 34% |
| Need for greater flexibility and accessibility | Students wanted easier navigation and review options. | Requests for back button, progress tracking, downloadable content. | Q1: 22% |
| Desire for additional explanations and resources | Students requested more explanations, examples, and links to resources. | “More explanation of the answers” | Q1: 9%; 13% |
